# Chronic Basal Ganglia Infarction With PCR‐Identified *Paeniclostridium sordellii*: A Rare Neuropathological Case Report

**DOI:** 10.1155/crip/7304864

**Published:** 2026-01-07

**Authors:** Cassandra Lamm, Angus Toland, Samuel Guzman

**Affiliations:** ^1^ Department of Pathology, University of Colorado, Anschutz Medical Center, Aurora, Colorado, USA, ucdenver.edu

## Abstract

**Background:**

Within 1 month of diagnosis, 40% of patients with positive blood cultures will develop CNS complications. Rare pathogens, that is, *Paeniclostridium sordellii*, pose diagnostic challenges due to atypical presentations and detection difficulties.

**Case Presentation:**

A 67‐year‐old man presented with metabolic encephalopathy and diabetes insipidus. Imaging showed bilateral basal ganglia abnormalities and an enhancing lesion near the anterior commissure, suspicious for neoplasm. Postmortem findings included reactive gliosis, atypical lymphocytic inflammation, and polyclonal plasma cells. NGS performed at the University of Washington confirmed *P. sordellii* infection.

**Neuropathologic Findings:**

The gross and histologic examination showed mild hydrocephalus, right hippocampal atrophy, and anterior commissure–centric inflammation. Vascular congestion, arteriosclerosis with microthrombi, and oligodendrocyte loss were present without hemorrhage.

**Differential Diagnosis:**

Hemophagocytic lymphohistiocytosis and CNS lymphoma were considered but ruled out microscopically.

**Conclusion:**

This case highlights the importance of considering *P. sordellii* in CNS inflammatory lesions and demonstrates the utility of molecular diagnostics in negative cultures.

## 1. Introduction

*Paeniclostridium sordellii* is a rare but highly virulent, spore‐forming, anaerobic, Gram‐positive bacterium. It is found in rural soil, the gastrointestinal tracts of animals and humans, and rarely in vaginal flora [1, 2]. Infections are uncommon but potentially fatal, especially in postpartum or postabortive women, intravenous drug users, and trauma/surgical patients. Clinical presentation often includes afebrile shock with tachycardia, hypotension, leukemoid reaction, hemoconcentration, edema, and hemorrhage*. P. sordellii* produces a lethal toxin, TcsL, which disrupts endothelial integrity via the SEMA6A and SEMA6B receptors, causing rapid systemic decompensation [3]. In animals, *P. sordellii* has been implicated in enteric and histotoxic disease in horses, ruminants, and birds, with pathology often demonstrating mucosal necrosis, hemorrhage, and systemic vascular injury [1]. Central nervous system inflammation and necrosis due to hemophagocytic lymphohistiocytosis (HLH) can pose a diagnostic challenge when a brain lesion contains a dense lymphohistiocytic infiltrate, especially in the absence of systemic findings [4], highlighting the need for a high index of suspicion and a broad differential when encountering unusual brain lesions.

## 2. Case Presentation

A 67‐year‐old man was found deceased at home following an eight‐day hospitalization for acute metabolic encephalopathy and severe hypernatremia with a subsequent eight‐day inpatient rehabilitation admission. Neurological deficits during his hospital stay included acute global debility, acute cognitive impairment, unsteady gait, and persistent blurry vision. His hospital course included suspected central diabetes insipidus and a presumed brain lesion. Imaging revealed bilateral basal ganglia T2 signal abnormalities, later evolving into an enhancing lesion near the anterior commissure. Laboratory results during hospitalization showed an elevated serum sodium (172 mmol/L) and an elevated plasma osmolality (358 mOsm/kg), with a low urine osmolality (157 mOsm/kg) and low urine specific gravity, 1.004, consistent with central diabetes insipidus. Cerebrospinal fluid collected during admission showed elevated white cells with a lymphocyte predominance (56%) and a protein of 157 units; cultures of the cerebrospinal fluid were negative. He was found deceased 15 days after discharge from inpatient rehabilitation. The decedent lived alone, and no information was provided regarding the decedent′s state of health after discharge. The postmortem interval from death to autopsy was 3 days. The brain was sent to the University of Colorado for neuropathologic examination as a brain‐only autopsy case.

## 3. Neuropathologic Findings

The brain weighed 1585 g in the fresh state and 1550 g in the postfixation state. Minimal decompositional changes were present. The dural concavities were shiny with no epidural or subdural hemorrhages. The cerebral leptomeninges were translucent and free of hemorrhage or exudate, though mild vascular congestion was present. The gyral configuration was normal without edema or cortical atrophy. The Circle of Willis and tributary arteries had a normal anatomic configuration with no evidence of atherosclerosis. Vertebral arteries were not available for examination. Cranial nerves were intact and symmetrical. The cerebellar tonsils and uncus were symmetric without necrosis.

Coronal sections of the cerebral hemispheres revealed mild to moderate hydrocephalus and dilation of the lateral ventricles, more prominent in the posterior horns. There was asymmetrical right hippocampal atrophy and right amygdala enlargement (Figure 1a). Bilateral substantia nigra showed decreased pigmentation.

Figure 1Gross photographs. (a) Right hippocampal atrophy and amygdala enlargement. (b) Anterior commissure lesion.

**Figure:**
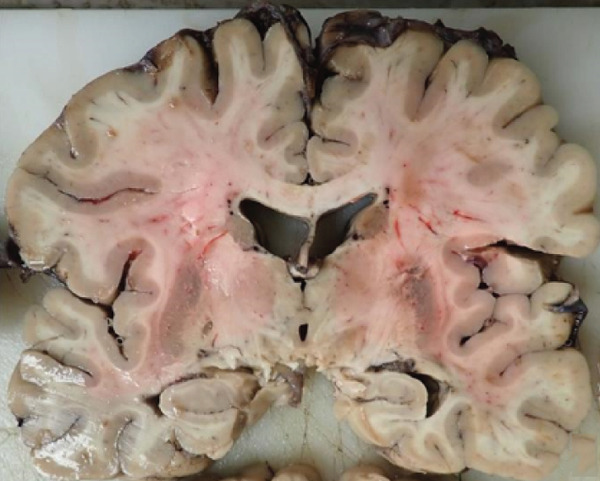
(a)

**Figure:**
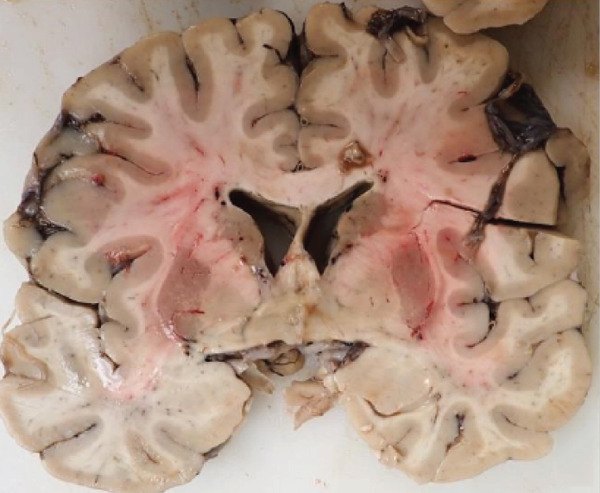
(b)

A cavitary, red‐brown lesion was present in the anterior commissure extending into the hypothalamus (Figure 1b). There was notable ventricular dilation and hippocampal atrophy. The leptomeninges demonstrated vascular congestion. Microscopic examination of the anterior commissure showed atypical lymphocytic inflammatory cells clustered around vasculature (Figure 2a) with associated reactive gliosis (Figure 3f). The neocortex, including regions of right frontal, right parietal, right temporal, and left occipital lobes, showed edema, chronic inflammation, moderate to severe small vessel arteriosclerosis with microthrombi, and decreased oligodendrocyte density. There was moderate to severe arteriosclerosis noted in the left basal ganglia and thalamus. The right hippocampus was atrophic and contained red neurons, indicative of ischemic injury. The cerebellum was preserved with intact foliar structure, granular layer, and Purkinje cells. The pons and midbrain exhibited mild edema with appropriate and decreased pigmentation of the locus coeruleus and substantia nigra, respectively, and no Lewy body pathology. The medulla was edematous. Increased corpora amylacea were present within the ventricular spaces. The choroid plexus showed calcifications. No hemorrhage was noted, and ischemic changes were limited to the right hippocampus.

Figure 2Microscopic photographs of right anterior commissure lesion. (a, b) H&E demonstrates perivascular and parenchymal lymphocytic infiltrates. (c) Numerous Mott cells are present, confirmed via (d) positive CD138 and (e, f) kappa/lambda immunohistochemical staining.

**Figure:**
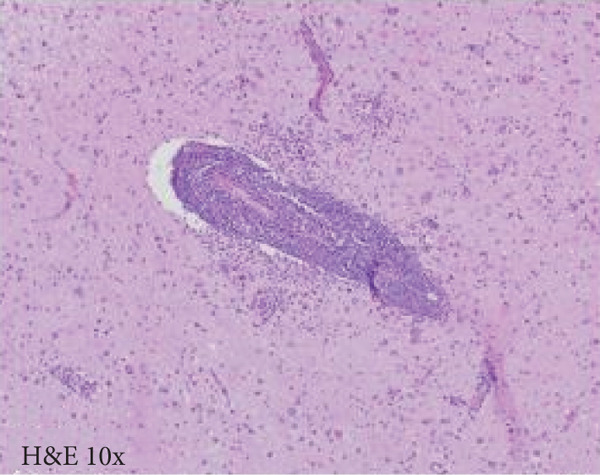
(a)

**Figure:**
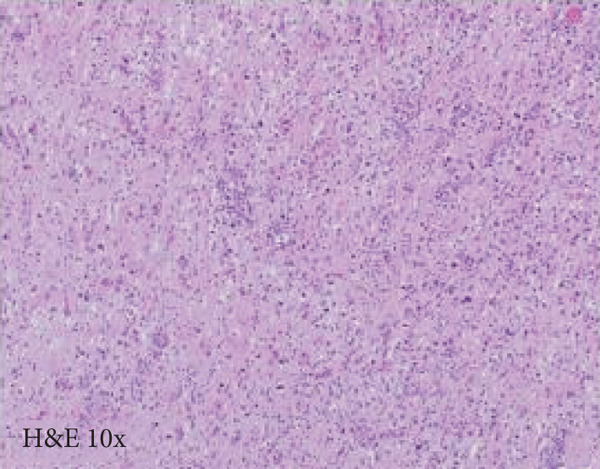
(b)

**Figure:**
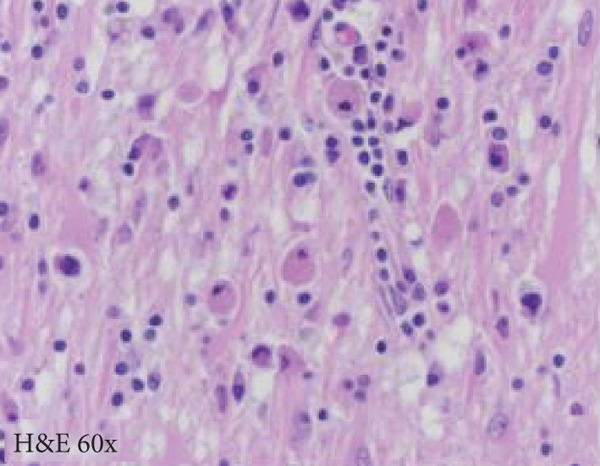
(c)

**Figure:**
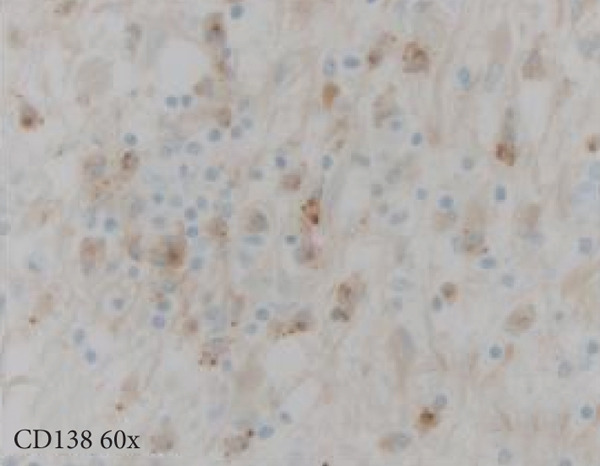
(d)

**Figure:**
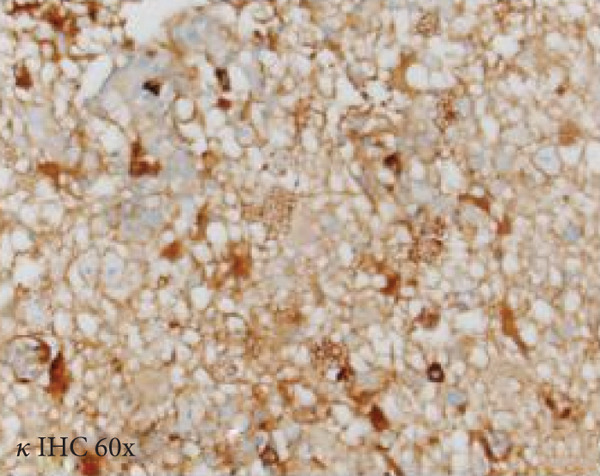
(e)

**Figure:**
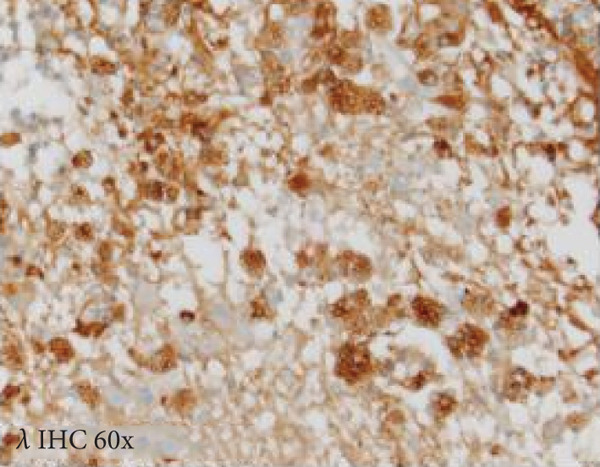
(f)

Figure 3Microscopic photographs of right anterior commissure lesion. The perivascular lymphocytic infiltrate is mixed with predominantly T cells, as highlighted by (a) CD3 staining, and a smaller population of (b) PAX5‐positive B cells. There are no intracytoplasmic red blood cells within Mott cells as demonstrated by the lack of (c) glycophorin and (d) CD163 staining. (e) Luxol fast blue combined with periodic acid‐Schiff (LFB/PAS) staining shows no plaque formation or demyelination. (f) GFAP staining is increased, highlighting reactive gliosis.

**Figure:**
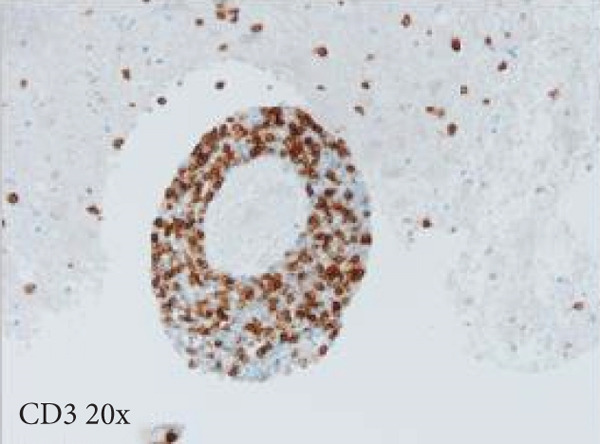
(a)

**Figure:**
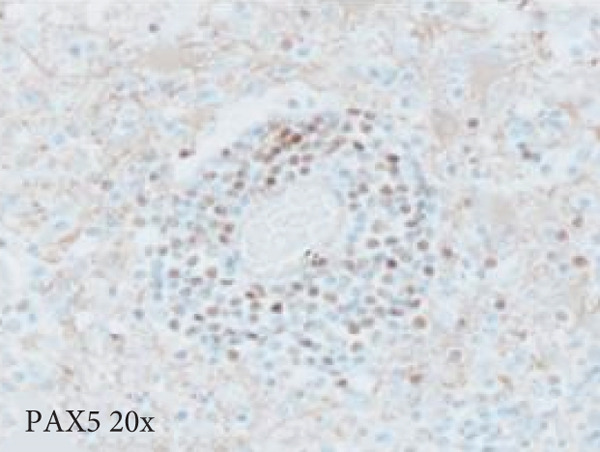
(b)

**Figure:**
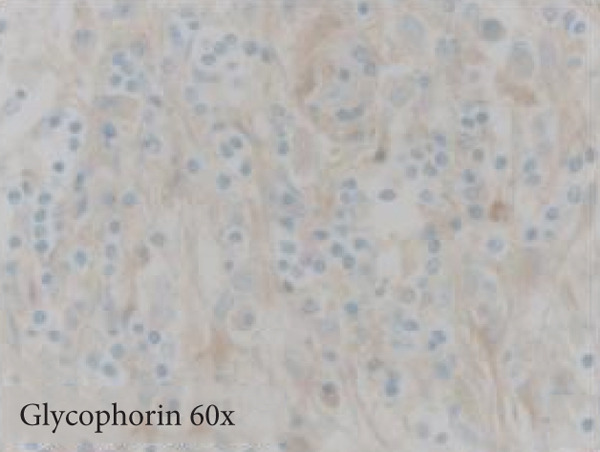
(c)

**Figure:**
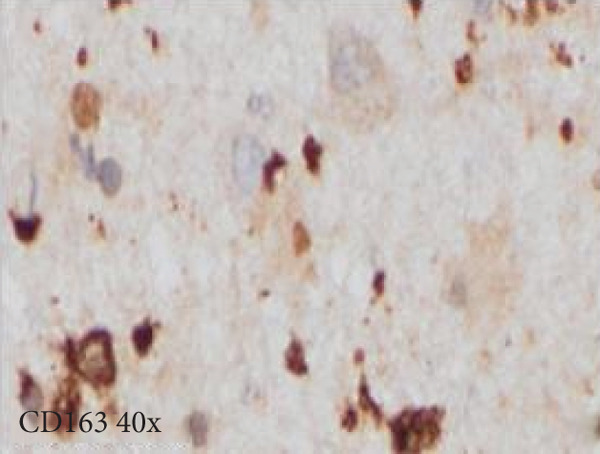
(d)

**Figure:**
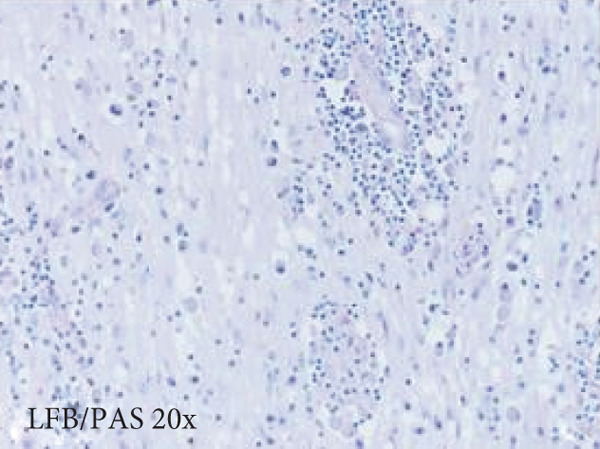
(e)

**Figure:**
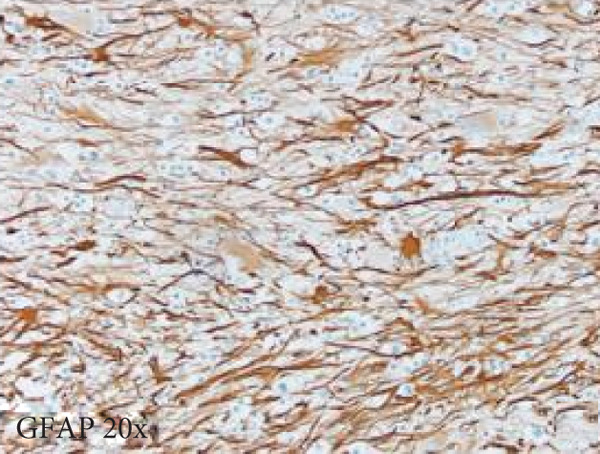
(f)

Microscopy also demonstrated perivascular and parenchymal lymphocytic infiltrates (Figure 2a,b), reactive gliosis, and focal necrosis. Numerous Mott cells (Figure 2c) were confirmed by CD138 and kappa/lambda immunohistochemical staining (Figures 2d, 2e, and 2f), indicative of a polyclonal plasma cell response. Immunohistochemistry showed mixed CD3‐positive T cells and CD20‐ and PAX5‐positive B cells (Figure 3a,b). Luxol fast blue combined with periodic acid‐Schiff (LFB/PAS) staining was performed, which revealed significant myelin disruption due to infarction without identification of an active demyelinating process (Figure 3e). Glial fibrillary acidic protein (GFAP) staining highlighted reactive gliosis (Figure 3f). Immunohistochemical and special staining for infectious agents were negative or nonspecific, with negative staining for GMS, PAS‐F, Fite, and *Toxoplasma* IHC and nonspecific Gram staining. Despite a lack of specific support from special stains and immunohistochemical stains, a likely infectious process was favored based on the results above along with the decedent′s clinical course, and a block of paraffin‐embedded tissue of the right anterior commissure lesion was sent to the University of Washington for bacterial, fungal, and toxoplasma universal PCR testing. Bacterial PCR 16S rRNA sequencing identified the presence of *P. sordellii*. Reflexive next‐generation sequencing (NGS) confirmed a minor population of this species, supporting the diagnosis. *Cutibacterium acnes* was also detected via NGS testing and interpreted as a likely contaminant.

### 3.1. Differential Diagnosis

The primary considerations were HLH and CNS lymphoma. Given the lymphocytic infiltrates surrounding blood vessels in the anterior commissure (Figure 2a), both diagnoses were strongly considered during initial microscopic evaluation. HLH can present with CNS inflammation, necrosis, and perivascular lymphocytes, making it challenging to distinguish from other inflammatory or neoplastic processes [4]. However, the absence of glycophorin‐positive hemophagocytosis (Figure 3c,d) on histology argued against HLH. CNS lymphoma was also considered due to the dense cellularity and angiocentric pattern of inflammatory infiltrate but was ultimately excluded by immunohistochemistry, which demonstrated a polyclonal population of B and T cells (Figure 3a,b), along with a marked polytypic plasma cell reaction confirmed by CD138 and mixed kappa/lambda staining (Figures 2d, 2e, and 2f). These findings supported a reactive rather than neoplastic process.

## 4. Discussion

This case illustrates an exceedingly rare CNS manifestation of *P. sordellii* infection. The pathogen′s lethal toxin, TcsL, binds semaphorin receptors (SEMA6A/SEMA6B), disrupting endothelial cells and inducing inflammation, edema, and hemorrhage [3]. Toxin‐mediated injury of this type has also been described in enteric infections of animals, where vascular injury and mucosal necrosis produce acute colitis and systemic decompensation [1, 2]. Lee et al. showed that the TcsL–SEMA6A interaction is mediated through a specific receptor‐binding domain, which can be competitively inhibited by the soluble ectodomain of SEMA6A, suggesting a potential therapeutic avenue. The patient′s chronic basal ganglia infarcts, polyclonal inflammatory response, and diabetes insipidus likely reflect a subacute‐to‐chronic manifestation of this toxin‐mediated injury. Diagnostic delays due to culture‐negative findings and nonspecific radiologic features further underscore the need for advanced molecular diagnostics. Our findings add to the emerging body of literature suggesting that *P. sordellii* should be considered in both systemic and organ‐specific infections, even in the absence of toxin gene expression or fulminant toxic shock. Toxin gene expression was not tested in this instance, and, due to the minimal information of the decedent′s health after discharge, it is difficult to assess if he ever was in toxic shock.

Some potential downfalls of this case include the possibility that, given the available resources, definitive diagnosis of the individual organism may not be accomplished. This is mainly due to rare contamination during the postmortem interval, and since no organisms are identified on representative sections, it is difficult to prove definitively that this infection was the direct cause. Uzal et al. discuss the possibility of identifying *P. sordellii* via PCR testing [5]; although this was done here, it is possible that the organisms were present on tissue sent for PCR testing and not present on histologic sections examined and stained. As discussed above, some individuals are colonized with *P. sordellii* although the most common location is the vaginal canal and nonapplicable in this case. Furthermore, the decedent was found deceased at home and not in association with a soil source, further arguing against postmortem environmental contamination. The literature on *P. sordellii* infections seems to correlate with the patient presentation and findings in this particular case, so it is unlikely that the positive molecular findings are primarily due to postmortem overgrowth. A full autopsy was not performed in this case, and the decedent′s health status in the days leading up to their demise is not documented, and so unfortunately, it is impossible to know if there were other signs or symptoms of a systemic bacterial infection in the immediate antemortem period. That being said, the main pathologic finding in this case was chronic infarction secondary to bacterial infection of the bilateral basal ganglia and focal necrotizing bacterial infection in the ventral medulla. Since the only pathologic bacterial that was found on PCR testing was *P. sordellii*, this is the most likely cause of the infection.

## 5. Conclusion

This case underscores the importance of including atypical infectious etiologies in the differential diagnosis of CNS lesions, particularly when standard testing is inconclusive. The findings support the utility of advanced molecular diagnostics and the relevance of recent mechanistic insights into *P. sordellii* virulence.

## Consent

Due to the patient′s death and removal of identifiable information, formal consent was not obtained.

## Conflicts of Interest

The authors declare no conflicts of interest.

## Funding

No funding was received for this manuscript.

## Data Availability

Data sharing is not applicable to this article as no datasets were generated or analyzed during the current study.
